# Electrochemical performance of Ni_*x*_Co_1-*x*_MoO_4 _(0 ≤ *x *≤ 1) nanowire anodes for lithium-ion batteries

**DOI:** 10.1186/1556-276X-7-35

**Published:** 2012-01-05

**Authors:** Kyung-Soo Park, Seung-Deok Seo, Hyun-Woo Shim, Dong-Wan Kim

**Affiliations:** 1 Department of Materials Science and Engineering, Ajou University, Suwon, 443-749, Republic of Korea

## Abstract

Ni_*x*_Co_1-*x*_MoO_4 _(0 ≤ *x *≤ 1) nanowire electrodes for lithium-ion rechargeable batteries have been synthesized via a hydrothermal method, followed by thermal post-annealing at 500°C for 2 h. The chemical composition of the nanowires was varied, and their morphological features and crystalline structures were characterized using field-emission scanning electron microscopy and X-ray powder diffraction. The reversible capacity of NiMoO_4 _and Ni_0.75_Co_0.25_MoO_4 _nanowire electrodes was larger (≈520 mA h/g after 20 cycles at a rate of 196 mA/g) than that of the other nanowires. This enhanced electrochemical performance of Ni_*x*_Co_1-*x*_MoO_4 _nanowires with high Ni content was ascribed to their larger surface area and efficient electron transport path facilitated by their one-dimensional nanostructure.

## Introduction

Among the types of anode materials available for rechargeable lithium-ion batteries, graphite has been commercialized. However, because of its drawbacks, such as capacity limitations (theoretical capacity of 372 mA h/g), initial loss of capacity, and structural deformation [1,2], one of the current areas of interest in lithium-ion battery research is the search for new anode candidates that have large reversible capacities. In the past decade, transition metal oxides (M_*x*_O_*y*_, M = Co, Ni, Cu, Fe) that can deliver a high reversible capacity by conversion reaction mechanisms (M_*x*_O_*y *_+ *ne*^- ^+ *n*Li^+ ^↔ *x*M^0 ^+ Li_*n*_O_*y*_) have been considered as an alternative to commercial graphite anodes for lithium-ion batteries [3-10].

Metal molybdates, AMoO_4_-type compounds (where A is a divalent metal ion), have attracted the interest of researchers because of their electronic and magnetic properties and their many applications, such as catalysis and photoluminescence [11-14]. Recently, CoMoO_4 _and NiMoO_4_, synthesized with nanowire morphology by a simple hydrothermal method, were exploited as materials for lithium-ion batteries but were only applied as cathodes [15,16].

In this paper, we report the fabrication of Ni_*x*_Co_1-*x*_MoO_4 _(0 ≤ *x *≤ 1) nanowire electrodes by a hydrothermal method, followed by thermal post-annealing. We also demonstrate the superior electrochemical performance of Ni_*x*_Co_1-*x*_MoO_4 _nanowires for lithium-ion battery anodes.

## Experimental details

Ni_*x*_Co_1-*x*_MoO_4 _(0 ≤ *x *≤ 1) nanowires were synthesized by a simple hydrothermal method, in which high purity Ni(NO_3_)_2_·6H_2_O (99.999%; Sigma-Aldrich, Saint Louis, MO, USA), Co(NO_3_)_2_·6H_2_O (98%; Sigma-Aldrich, Saint Louis, MO, USA), and Na_2_MoO_4_·2H_2_O (99.5%; Sigma-Aldrich, Saint Louis, MO, USA) were used as source materials, followed by post-annealing at an elevated temperature. Initially, to prepare a clean solution (total cationic concentration of 0.1 M) with a molar fraction (*x*) of Ni (*x *= 0, 0.25, 0.5, 0.75, and 1), controlled amounts of Co- and Ni-containing reagents were dissolved in deionized water (80 mL) under constant magnetic stirring; then, the solution was added to an aqueous solution (80 mL) containing 0.1 M of Na_2_MoO_4_·2H_2_O. This resulting solution was transferred into a Teflon-lined stainless steel autoclave, sealed, and maintained at 180°C for 8 h. After the reaction was completed, the resulting solid products were harvested by centrifugation, washed with deionized water and acetone several times, and then dried at 60°C for 6 h in a vacuum oven. Finally, the as-prepared hydrate nanowire precursors were post-annealed at 500°C for 2 h to dehydrate them.

The morphologies and crystal structures of the prepared Ni_*x*_Co_1-*x*_MoO_4_·*n*H_2_O and Ni_*x*_Co_1-*x*_MoO_4 _(0 ≤ *x *≤ 1) nanowires were investigated using field-emission scanning electron microscopy [FE-SEM] (10 kV; FEI NOVA, Tokyo, Japan) and X-ray powder diffraction [XRD] (*λ*_CuKa _= 1.5405 Å; Miniflex II, Rigaku, Tokyo, Japan). The thermal behavior of the as-prepared hydrate samples was analyzed by thermogravimetric analysis [TGA] (DTG-60H, Shimadzu, Kyoto, Japan). For TGA, the samples were heated from room temperature up to 800°C at a heating rate of 10°C/min in air.

For the electrochemical evaluation of the Ni_*x*_Co_1-*x*_MoO_4 _(0 ≤ *x *≤ 1) nanowires, positive electrode films were cast on a Cu foil by mixing each nanowire powder (1 to 2 mg) with Super P carbon black (MMM Carbon, Brussels, Belgium) and Kynar 2801 binder (PVdF-HFP, Arkema Inc., King of Prussia, PA, USA) in a mass ratio of 70:15:15. The assembled Swagelok-type cells composed of a positive electrode, negative electrode (lithium metal-foil), and separator film (Celgard 2400, Celgard LLC, Charlotte, NC, USA) saturated with a liquid electrolyte consisting of LiPF_6 _(1 M) dissolved in a solution of ethylene carbonate and dimethyl carbonate (1:1 *v*/*v*) were cycled at voltages between 0.01 and 3.0 V using an automatic battery cycler (WBCS 3000, WonaTech, Seoul, South Korea).

## Results and discussion

**Figure 1:**
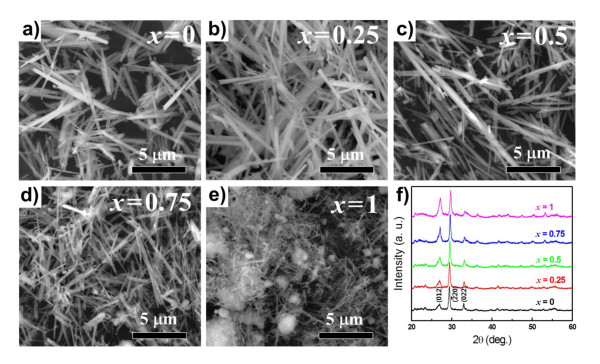
**Morphologies and crystal structures of Ni_*x*_Co_1-*x*_MoO_4_·*n*H_2_O nanowires**. (**a-e**) Typical FE-SEM images of as-prepared Ni_*x*_Co_1-*x*_MoO_4_·*n*H_2_O nanowire precursors with various *x *values and (**f**) their corresponding X-ray diffraction patterns (by KS Park et al.).

Figures 1a to 1e show typical FE-SEM images of as-prepared Ni_*x*_Co_1-*x*_MoO_4_·*n*H_2_O precursors with various cationic compositions. All of the samples were of nanowire morphology regardless of their compositional differences. However, the diameters and lengths of the nanowires tended to decrease with increasing Ni concentration. As shown in Figure 1e, the NiMoO_4_·*n*H_2_O nanowires were agglomerated acutely and even formed clusters that were several microns in size. The agglomeration of the nanowires might be due to strong attractive forces between the small-sized nanowire particles. The crystalline structures of the as-prepared Ni_*x*_Co_1-*x*_MoO_4_·*n*H_2_O nanowires were confirmed from their XRD patterns, as seen in Figure 1f. The XRD patterns taken from all Ni_*x*_Co_1-*x*_MoO_4_·*n*H_2_O nanowires agreed well with previously reported patterns for CoMoO_4_·*n*H_2_O (JCPDS no.: 26-0477).

**Figure 2:**
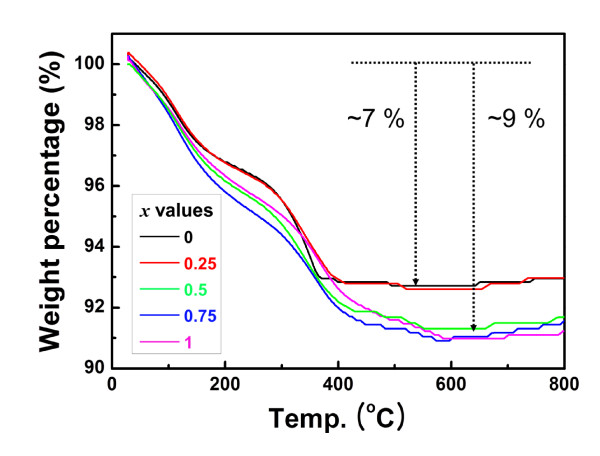
**Thermal behavior of Ni_*x*_Co_1-*x*_MoO_4_·*n*H_2_O nanowires**. TG curves for as-prepared Ni_*x*_Co_1-*x*_MoO_4_·*n*H_2_O nanowire precursors with various values of *x*, heating at a rate of 10°C/min in air (by KS Park et al.).

The dehydration process was investigated using the thermogravimetric [TG] technique; the results of which are shown in Figure 2. The typical TG curves obtained from the Ni_*x*_Co_1-*x*_MoO_4_·*n*H_2_O nanowires in the temperature range from 30°C to 800°C showed net weight losses of 7% to 9% for each sample. This weight loss was mainly attributed to the evolution of species related to water molecules, such as reversibly bound water molecules (low temperature), water molecules forming an integral part of the crystal structure of Ni_*x*_Co_1-*x*_MoO_4_·*n*H_2_O (medium temperature), and water molecules reversibly bound to the hydrate crystal phase (high temperature) [14,15]. On the basis of the TG results, the post-annealing temperature for all the hydrate samples was 500°C, at which almost all water molecules were removed sufficiently to form Ni_*x*_Co_1-*x*_MoO_4 _nanowires.

**Figure 3:**
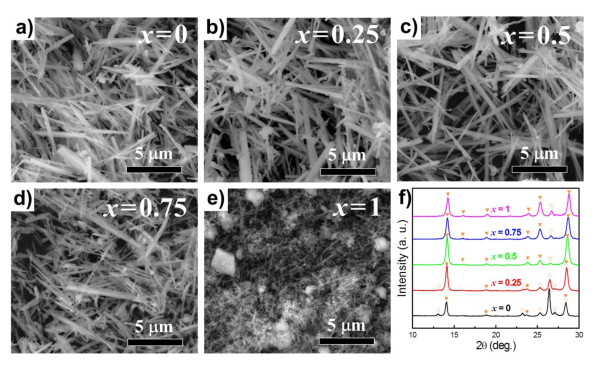
**Morphologies and crystal structures of Ni_*x*_Co_1-*x*_MoO_4 _nanowires**. (**a-e**) FE-SEM images of Ni_*x*_Co_1-*x*_MoO_4 _nanowires with various *x *values and (**f**) their corresponding X-ray diffraction patterns. Filled circle, empty circle, filled inverted triangle, and empty inverted triangle correspond to α-CoMoO_4_, β-CoMoO_4_, α-NiMoO_4_, and β-NiMoO_4_, respectively (by KS Park et al.).

Figures 3a to 3e show FE-SEM images of Ni_*x*_Co_1-*x*_MoO_4 _(0 ≤ *x *≤ 1) nanowires obtained by post-annealing the Ni_*x*_Co_1-*x*_MoO_4_·*n*H_2_O nanowire precursors at 500°C for 2 h. All post-annealed samples maintained their pristine nanowire morphologies. Although a marked change in the diameter or length of the nanowires after post-annealing was not observed, they seemed to be agglomerated slightly and were forming bundles, which could lead to a decrease in their surface area. In order to confirm the change in surface area that may have occurred during post-annealing of Ni_*x*_Co_1-*x*_MoO_4_·*n*H_2_O nanowire precursors, the Brunauer-Emmett-Teller [BET] (Belsorp-mini, BEL Japan Inc., Osaka, Japan) technique was carried out at liquid nitrogen temperature. From the results presented in Table 1 it can be confirmed that the surface areas of Ni_*x*_Co_1-*x*_MoO_4 _nanowires in all compositions were slightly decreased compared with those of the corresponding Ni_*x*_Co_1-*x*_MoO_4_·*n*H_2_O nanowires. This was ascribed to the aggregation and growth of the nanowires. Furthermore, it was found that the surface areas of the nanowires gradually increased with increasing Ni concentration because of the corresponding decrease in their diameter and length.

**Table 1:** Surface areas of prepared samples

	Ni_*x*_Co_1-*x*_MoO_4_·*n*H_2_O	Ni_*x*_Co_1-*x*_MoO_4_
*x *value	Surface area (m^2^/g)	
0	9.5	7.8
0.25	10.1	9.0
0.5	12.7	10.3
0.75	21.0	18.6
1	47.4	39.2

Comparison of surface areas for as-prepared Ni_*x*_Co_1-*x*_MoO_4_·*n*H_2_O and Ni_*x*_Co_1-*x*_MoO_4 _nanowires dehydrated at 500°C for 2 h (by KS Park et al.).

To investigate the crystal structures of the Ni_*x*_Co_1-*x*_MoO_4 _nanowires, their XRD patterns were examined carefully. The XRD patterns obtained for the CoMoO_4 _and NiMoO_4 _nanowires in Figure 3f corresponded well with their bulk materials (JCPDS nos.: 25-1434, 21-0868, 33-0948, and 45-0142 for α-CoMoO_4_, β-CoMoO_4_, α-NiMoO_4_, and β-NiMoO_4_, respectively). However, as can be seen in Figure 3f, both end-members had α- and β-phases corresponding to each material together. It is known that single-phase β-CoMoO_4 _and α-NiMoO_4 _can be formed by fast cooling and by slow cooling, respectively, to room temperature after post-annealing of their hydrates [14,17,18]. Meanwhile, D. Vie et al. reported that the α-CoMoO_4 _phase became detectable as a minority phase after heat treatment of the amorphous solid precursor at a temperature above 700°C [17]. On the basis of these reports, we believe that the coexistence of α- and β-phases in CoMoO_4 _and NiMoO_4 _nanowires can be attributed to the medium cooling rate (≈10°C/min to 30°C/min) applied after post-annealing or to a change of phase transition temperature with the unique morphologies of the samples.

Among the Ni_*x*_Co_1-*x*_MoO_4 _nanowires with a medium composition, the XRD pattern of the Ni_0.25_Co_0.75_MoO_4 _nanowires agreed well with that of CoMoO_4_. However, in contrast with that observed for pure CoMoO_4_, the peaks corresponding to α-CoMoO_4 _became more dominant compared with those of β-CoMoO_4_, indicating that relative intensities of the diffraction peaks of the α-phase increase with Ni content [17]. In the case of the Ni_0.5_Co_0.5_MoO_4 _nanowires, it was found that the XRD pattern corresponded closer with pure NiMoO_4 _than with pure CoMoO_4 _(Figure 3f). This implies that the basic crystal structure of the nanowires was transformed from CoMoO_4_-related structures into NiMoO_4_-related structures. The XRD pattern of the Ni_0.75_Co_0.25_MoO_4 _nanowires also agreed with that of pure NiMoO_4_. It should be noted that no secondary phases, such as NiO and CoO, were detected in any of the XRD patterns, which indicated that CoMoO_4 _and NiMoO_4 _formed solid solutions perfectly.

In general, most reports on the lithium reactivity of molybdates have been focused on their application as anode materials for lithium-ion batteries because they could deliver high reversible capacity through a conversion reaction with lithium [19-21]. Moreover, NiO and CoO have also been known as anode materials, reacting with lithium by an equivalent mechanism with a molybdate [3]. On the basis of such knowledge, we expected the following electrochemical reaction mechanism of Ni_*x*_Co_1-*x*_MoO_4 _with lithium:

(1) $$\text{N}{\text{i}}_{x}\text{C}{\text{o}}_{\text{1}-x}\text{Mo}{\text{O}}_{\text{4}}+\text{ 8L}{\text{i}}^{+}+\text{ 8}e^{-}\to x\text{Ni }+\;\left(\text{1}-x\right)\text{Co }+\text{ Mo }+\text{ 4L}{\text{i}}_{\text{2}}\text{O}$$

(2) $$x\text{Ni }+\;\left(\text{1}-x\right)\text{Co }+\text{ Mo }+\text{ 4L}{\text{i}}_{\text{2}}\text{O}↔x\text{NiO }+\;\left(\text{1}-x\right)\text{CoO }+\text{ Mo}{\text{O}}_{\text{3}}+\text{ 8L}{\text{i}}^{+}+\text{ 8}e^{-}$$

**Figure 4:**
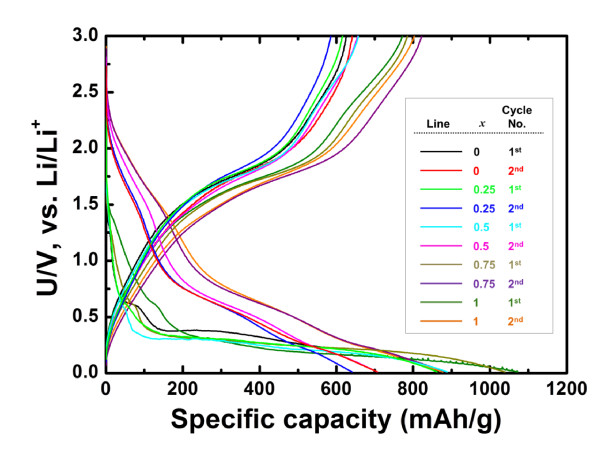
**Charging-discharging curves of Ni_*x*_Co_1-*x*_MoO_4 _nanowire electrodes**. Charging-discharging curves of the Ni_*x*_Co_1*-x*_MoO_4 _nanowire electrodes with various *x *values at a rate of C/5 (by KS Park et al.).

To evaluate the electrochemical performance of the Ni_*x*_Co_1-*x*_MoO_4 _nanowire electrodes, typical voltage-specific capacity curves were recorded at a current rate of C/5 (≈196 mA/g; note that 1 C ≈ 980 mA/g on the basis of the reaction in Equation 2) in a potential window between 0.01 and 3.0 V (Figure 4). In the first discharge reaction, samples containing nickel reacted with lithium at a lower potential region (≈0.4 V to 0.01 V) compared with the CoMoO_4 _nanowire electrode (≈0.3 V to 0.01 V). Notably, the first discharge capacities for Ni_0.75_Co_0.25_MoO_4 _and NiMoO_4 _were higher than their theoretical capacities; this might be ascribed to the formation of the solid electrolyte interface-like organic layer. Subsequent second discharge-charge reactions occurred reversibly on the basis of the reaction in Equation 2. These highly reversible capacities of Ni_*x*_Co_1-*x*_MoO_4 _nanowire electrodes could be an evidence for delivering capacity by conversion mechanism.

**Figure 5:**
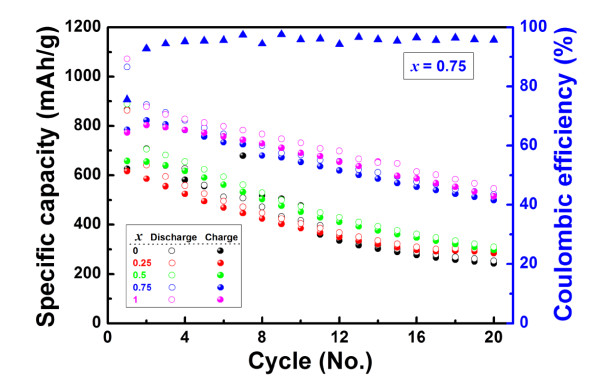
**Electrochemical properties of Ni_*x*_Co_1-*x*_MoO_4 _nanowire electrodes**. Variation of the discharge (open circles)-charge (solid circles) specific capacity and coulombic efficiency (solid triangle) versus the cycle number at a rate of C/5 for the Ni_*x*_Co_1-*x*_MoO_4 _nanowire electrodes with various *x *values (by KS Park et al.).

Figure 5 shows the specific capacity of Ni_*x*_Co_1-*x*_MoO_4 _nanowire electrodes versus the cycle number at a current rate of C/5. When the values of *x *were 0, 0.25, and 0.5, the reversible capacities of Ni_*x*_Co_1-*x*_MoO_4 _nanowire electrodes faded rapidly even during the initial cycles, resulting in a poor reversible capacity of ≈290 mA h/g after 20 cycles. This result was attributed to the small surface areas of the nanowire electrodes. From the BET results summarized in Table 1 it was found that their surface areas were very small compared with those of the other nanowires, which led to a small electrode/electrolyte interface, resulting in a poor reversible capacity. Meanwhile, the reversible capacity of the Ni_*x*_Co_1-*x*_MoO_4 _nanowire electrodes with *x *values of 0.75 and 1 (≈520 mA h/g after 20 cycles) was larger than that of the previously mentioned three nanowire electrodes because of the increased electrode/electrolyte interface that resulted from the larger surface area of the nanowires (Table 1). In accordance with the BET results, the electrochemical performance of the NiMoO_4 _nanowire electrode was expected to be vastly superior compared with that of the Ni_0.75_Co_0.25_MoO_4 _nanowire electrode. However, the electrochemical performances of both electrodes were similar, as shown in Figure 5 due to the aggregation of the NiMoO_4 _nanowires. When we fabricated the positive electrode, the carbon black that is commonly used as a conducting additive was mechanically mixed with the nanowires as the active material. At that time, clusters formed by aggregation of the NiMoO_4 _nanowires could not be mixed efficiently with carbon black, which resulted in impairing their electrochemical performance by restriction of the electronic conduction paths from each nanowire to a current collector. In contrast, the Ni_0.75_Co_0.25_MoO_4 _nanowires did not form such clusters during the synthesis process. For this reason, the Ni_0.75_Co_0.25_MoO_4 _nanowire electrode could deliver a reversible capacity as high as the NiMoO_4 _nanowire electrode. Furthermore, the Ni_0.75_Co_0.25_MoO_4 _nanowire electrode exhibited a superior coulombic efficiency of ≈95% (Figure 5).

## Conclusion

In summary, we have successfully synthesized Ni_*x*_Co_1-*x*_MoO_4 _(0 ≤ *x *≤ 1) nanowire electrodes by employing a hydrothermal method to prepare hydrate nanowires, followed by post-annealing of the as-prepared hydrate nanowires. Although α- and β-phases coexisted in all of the nanowire samples, no secondary phases were detected, indicating that CoMoO_4 _and NiMoO_4 _formed perfect solid solutions. Compared with the reversible capacity (≈290 mA h/g after 20 cycles) of the Ni_*x*_Co_1*-x*_MoO_4 _nanowire electrodes with *x *values of 0, 0.25, and 0.5, the reversible capacity of the nanowire electrodes containing a higher Ni content (*x *= 0.75 and 1) increased more (≈520 mA h/g after 20 cycles). Despite the difference in surface area between the NiMoO_4 _and Ni_0.75_Co_0.25_MoO_4 _nanowires, the reason for the observation of their similar reversible capacities was because of the agglomeration of NiMoO_4 _nanowires and the resulting restriction of the electronic conduction paths from each nanowire to a current collector. We anticipate that this approach will facilitate the tailoring of other electrodes based on solid solution materials, to provide electrodes with superior electrochemical performance.

## Competing interests

The authors declare that they have no competing interests.

## Authors' contributions

K-SP carried out the Ni_*x*_Co_1-*x*_MoO_4 _(0 ≤ *x *≤ 1) nanowire sample preparation and drafted the manuscript. S-DS and H-WS participated in microstructural and electrochemical analyses. D-WK designed the study, led the discussion of the results, and participated in writing the manuscript. All authors read and approved the final manuscript.

## References

[B1] Disma F Aymard L Dupont L Tarascon JM Effect of mechanical grinding on the lithium intercalation process in graphites and soft carbons J Electrochem Soc 1996 143 3959 10.1149/1.1837322

[B2] Disma F Lenain C Beaudoin B Aymard L Tarascon JM Unique effect of mechanical milling on the lithium intercalation properties of different carbons Solid State Ionics 1997 98 145 10.1016/S0167-2738(97)00108-2

[B3] Poizot P Laruelle S Grugeon S Dupont L Tarascon JM Nano-sized transition-metal oxides as negative-electrode materials for lithium-ion batteries Nature 2000 407 496 10.1038/35035045 11028997

[B4] Taberna PL Mitra S Poizot P Simon P Tarascon JM High rate capabilities Fe_3_O_4_-based Cu nano-architectured electrodes for lithium-ion battery applications Nat Mater 2006 5 567 10.1038/nmat1672 16783360

[B5] Kim DW Ko YD Park JG Kim BK Formation of lithium-driven active/inactive nanocomposite electrodes based on Ca_3_Co_4_O_9 _nanoplates Angew Chem Int Ed 2007 46 6654 10.1002/anie.200701628 17663491

[B6] Shim HW Jin YH Seo SD Lee SH Kim DW Highly reversible lithium storage in *Bacillus subtilis*-directed porous Co_3_O_4 _nanostructures ACS Nano 2011 5 443 10.1021/nn1021605 21155558

[B7] Li Y Tan B Wu Y Mesoporous Co_3_O_4 _nanowire arrays for lithium ion batteries with high capacity and rate capability Nano Lett 2008 8 265 10.1021/nl0725906 18072799

[B8] Shim HW Cho IS Hong KS Cho WI Kim DW Li electroactivity of iron (II) tungstate nanorods Nanotechnology 2010 21 465602 10.1088/0957-4484/21/46/465602 20972323

[B9] Lee GH Park JG Sung YM Chung KY Cho WI Kim DW Enhanced cycling performance of Fe^0^/Fe_3_O_4 _nanocomposite electrode for lithium-ion batteries Nanotechnology 2009 20 295205 10.1088/0957-4484/20/29/295205 19567958

[B10] Ko YD Kang JG Choi KJ Park JG Ahn JP Chung KY Nam KW Yoon WS Kim DW High rate capabilities induced by multi-phasic nanodomains in iron-substituted calcium cobaltite electrodes J Mater Chem 2009 19 1829 10.1039/b817120c

[B11] Liu J Huang X Li Y Li Z A general route to thickness-tunable multilayered sheets of sheelite-type metal molybdate and their self-assembled films J Mater Chem 2007 17 2754 10.1039/b703552g

[B12] Vilminot S André G Kurmoo M Magnetic properties and magnetic structure of Cu^II^_3_Mo^VI^_2_O_9_ Inorg Chem 2009 48 2687 10.1021/ic802410p 19267511

[B13] Chu WG Wang HF Guo YJ Zhang LN Han ZH Li QQ Fan SS Catalyst-free growth of quasi-aligned nanorods of single cryatal Cu_3_Mo_2_O_9 _and their catalytic properties Inorg Chem 2009 48 1243 10.1021/ic801885c 19128151

[B14] Rodriguez JA Chaturvedi S Hanson JC Albornoz A Brito JL Electronic properties and phase transformations in CoMoO_4 _and NiMoO_4_: XANES and time-resolved synchrotron XRD studies J Phys Chem B 1998 102 1347 10.1021/jp972137q

[B15] Xiao W Chen JS Li CM Xu R Lou XW Synthesis, characterization, and lithium storage capability of AMoO_4 _(A = Ni, Co) nanorods Chem Mater 2010 22 746 10.1021/cm9012014

[B16] Ding Y Wan Y Min YL Zhang W Yu SH General synthesis and phase control of metal molybdate hydrates MMoO_4_•nH_2_O (M = Co, Ni, Mn, n = 0, 3/4, 1) nano/microcrystals by a hydrothermal approach: magnetic, photocatalytic, and electrochemical properties Inorg Chem 2008 47 7813 10.1021/ic8007975 18681424

[B17] Vie D Martínez E Sapiña F Folgado JV Beltrán A Freeze-dried precursor-based synthesis of nanostructured cobalt-nickel molybdates Co_1-x_Ni_x_MoO_4_ Chem Mater 2004 16 1697 10.1021/cm035079w

[B18] Rodriguez JA Chaturvedi S Hanson JC Brito JL Reaction of H_2 _and H_2_S with CoMoO_4 _and NiMoO_4_: TPR, XANES, time-resolved XRD, and molecular-orbital studies J Phys Chem B 1999 103 770 10.1021/jp983115m

[B19] Kim SS Ogura S Ikuta H Uchimoto Y Wakihara M Reaction mechanisms of MnMoO_4 _for high capacity anode material of Li secondary battery Solid State Ionics 2002 146 249 10.1016/S0167-2738(01)01013-X

[B20] Leroux F Goward GR Power WP Nazar LF Understanding the nature of low-potential Li uptake into high volumetric capacity molybdenum oxides Electrochem Solid-State Lett 1998 1 255

[B21] Sharma N Shaju KM Rao GVS Chowdari BVR Dong ZL White TJ Carbon-coated nanophase CaMoO_4 _as anode material for Li ion batteries Chem Mater 2004 16 504 10.1021/cm0348287

